# Folic acid supplementation: what is new? Fetal, obstetric, long-term benefits and risks

**DOI:** 10.4155/fsoa-2015-0015

**Published:** 2016-04-21

**Authors:** Hind N Moussa, Susan Hosseini Nasab, Ziad A Haidar, Sean C Blackwell, Baha M Sibai

**Affiliations:** 1 Division of Maternal-Fetal Medicine, Department of Obstetrics, Gynecology & Reproductive Sciences, The University of Texas Health Science Center at Houston, Houston, TX, USA

**Keywords:** adverse pregnancy outcomes, folic acid, pre-conception supplement

## Abstract

The association between folic acid supplementation, prior to conception and/or during pregnancy and pregnancy outcomes, has been the subject of numerous studies. The worldwide recommendation of folic acid is at least 0.4 mg daily for all women of reproductive age, and 4–5 mg in high-risk women. In addition, evidence shows that folic acid supplementation could modulate other adverse pregnancy outcomes, specifically, in pregnancies complicated by seizure disorders, preeclampsia, anemia, fetal growth restriction and autism. This review summarizes the available national and international guidelines, concerning the indications and dosage of folic acid supplementation during pregnancy. In addition, it describes the potential preventive benefits of folic acid supplementation on multiple maternal and fetal outcomes, as well as potential risks.

**Figure 1.**
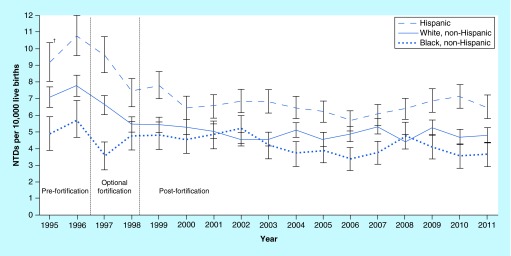
**Prevalence of neural tube defects (anencephaly and spina bifida) before and after mandatory folic acid fortification, by maternal race/ethnicity – 19 population-based birth defects surveillance programs, USA, 1995–2011.** Contributing programs are based in Arkansas, Arizona, California, Colorado, Georgia, Illinois, Iowa, Kentucky, Maryland, New Jersey, New York, North Carolina, Oklahoma, Puerto Rico, South Carolina, Texas, Utah, West Virginia and Wisconsin. ^†^95% confidence interval. NTD: Neural tube defect. Taken from Center for Disease Control and Prevention [7].

## Folic acid & neural tube defects

Neural tube defects (NTDs) are congenital abnormalities of the brain and spinal column that cause serious mortality and morbidity. In USA, NTDs affect 3000 pregnancies annually [1]. A third of these pregnancies are lost spontaneously or electively terminated.

The molecular requirements for neural tube closure are complex. Neural development occurs early in embryogenesis (by 6 weeks of gestation), when the majority of women are not aware of their pregnancy. Additionally, approximately 50% of pregnancies in USA are unplanned.

Folic acid, a water-soluble vitamin (B9), contributes to neural tube closure by enhancing cellular proliferation. It is an essential cofactor in folate-mediated one-carbon metabolism and in the epigenetic regulation of the transcription of genes that control neural closure [2].

The protective effect of folic acid supplementation is high when it is used preconceptionally, starting 1 month before conception and continuing until the end of the first trimester. Although women of reproductive age could benefit from a folate-rich diet, diet alone is not enough to increase the serum folate level and supplemental folic acid must be advised.

In 1991, the UKs Medical Research Council Vitamin Study Research Group conducted a randomized double-blind controlled study in seven countries [3]. The study population included 1195 pregnant women. They divided them in four groups, folic acid, other vitamins (A, D, B1, B2, B6, C and nicotinamide), both or neither. The folic acid intake was 4 mg preconceptionally. They detected a 72% reduction in recurrent risk in the incidence of NTDs in folate groups.

In 1992, the US Public Health Service (PHS) recommended that all women who are capable of becoming pregnant take 400 μg of folic acid daily as a supplement to prevent NTDs [4].

Studies revealed that the American diet for women of reproductive age did not contain folic acid rich products [5]. The folic acid intake of this group was not high enough to achieve the PHS recommendation. Thus, in 1998, mandatory fortification of cereal grain products with 140 μg of folic acid per 100 g was implemented [6]. Initially; a significant reduction in the rate of NTDs after the fortification was noted. Data from the prefortification period (1995–1996) and the postfortification period (biennial from 1999 to 2008, last 3 years of available data from 2009 to 2011 and all years from 1999 to 2011) were compared in terms of NTDs rate [7]. Based on this analysis, the Centers for Disease Control and Prevention (CDC) reported a reduction in the birth prevalence of spina bifida and anencephaly by 28% (using data from all participating programs), 35% (among programs with prenatal ascertainment) and 21% (among programs without prenatal ascertainment) after the fortification (Figure 1) [7].

Furthermore, in 2007, the CDC reported a significant reduction in serum and blood folate concentrations in women of reproductive age based on National Health and Nutrition Examination Survey (NHANES) data from surveys throughout 1999 to 2004 [8]. Two main factors could explain such a reduction: the reduction in fortification levels and the frequent use of low-carbohydrate and gluten-free diets [9], which are deficient in fortified food [10]. In 2009, an update released by the US Preventive Services Task Force (USPSTF) recommended that women planning of becoming pregnant should take a daily supplement containing 0.4–0.8 mg (400–800 μg) of folic acid [11].

Despite the introduced mandatory fortification of flour with folic acid in USA, this is not an established practice in the rest of the world, mainly Europe. Concerns about risks associated with folic acid metabolism led to several studies including one in Ireland, where fortification by manufacturers is voluntary [12]. In that study, the researchers took blood samples from both adults and newborns, and tested for the unmetabolized form of folic acid. The results showed that many people were already getting folic acid through their daily diet. A small proportion of their total folate was unmetabolized, suggesting it was in excess. They concluded that excess folic acid might increase the risk of cancer and mask some types of anemia [12].

Similarly, quantifying the effect of folic acid has always been the center of interest and research. In a review published in *Lancet* in 2001, 13 studies were evaluated [13]. It determined that an increase of 0.4 mg folic acid intake per day would reduce the risk of NTDs by approximately 36%, an increase of 1 mg per day would reduce the risk of NTDs by approximately 57%, and that an intake of 5 mg folic acid daily would reduce the risk of NTDs by approximately 85%. Therefore, they recommended increasing the folic acid intake for women planning a pregnancy from 0.4 mg to 5 mg tablets [13].

Current recommendations from the American College of Obstetricians and Gynecologist (ACOG), Royal College of Obstetricians and Gynecologists (RCOG) and Society of Obstetricians and Gynecologists of Canada (SOGC), are summarized in Table 1, 2 & 3, respectively.

Furthermore, folic acid supplementation could play a beneficial role on pregnancy outcomes that goes beyond the NTD reduction, such as in women with seizure disorder, preeclampsia, fetal growth restriction and future autism risk.

## Folic acid & anti epileptic drugs in pregnancy

The estimated incidence of seizure in pregnancy is around 3–5/1000 births in a year in USA, with more than 20,000 infants born to affected women [18,19]. Antiepileptic drugs (AED) have shown teratogenic effects and the rate of teratogenenesis caused by phenytoin is approximately 0.7–7% [20,21], with fetal hydantoin syndrome as pathognomonic. Carbamazepine (CBZ) has the teratogenicity of 2–6% [20,21], and is most commonly associated with cardiac malformations. Valporic acid (VPA), has the highest risk of teratogenesis, 7–12%, and could increase the risk of neural tube defect up to 1–2% [20].

Thus, folic acid supplementation is an important part of the prenatal care of women with epilepsy. Interference of AEDs in folate metabolism is mainly noted in women taking CBZ and VPA. In 1982, Robert *et al*. revealed the association between the CBZ and VPA and NTDs [22]. Several subsequent studies noted the association of CBZ exposure *in utero* and NTDs [23–25].

Due to the above-mentioned side effect of AEDs, experts suggest higher dosage of folic acid as a preconceptional supplement to prevent NTDs. The current international recommendations for patients who are taking AEDs are shown in Table 4.

## Folic acid & preeclampsia

In 2008, Wen *et al*. described the association between supplementation with multivitamins containing folic acid in the second trimester and reduced risk of preeclampsia [29]. Several mechanisms were investigated to explain such reduction.

Homocysteine was proposed to be an independent risk factor for developing gestational hypertension or preeclampsia [30–37]. High serum or plasma homocysteine has been detected in women with these disorders in antepartum or postpartum. Folic acid could correct hyperhomocysteinemia by optimizing the homocysteine pathway [38–41], thus can play a role in reduction of the incidence of gestational hypertension or preeclampsia. However, controversy over this still exists. Studies in USA and Canada have supported this hypothesis showing a reduction in the risk of gestational hypertension and preeclampsia by folic acid supplementation [29,42–43]. In contrast, studies in China [44] and Holland [45] did not show a significant reduction. To date, no international recommendation is available.

## Pregnancy-induced folic acid deficiency anemia

Anemia is one of the common complications in pregnancy. The most common type of anemia is iron deficiency anemia, followed by megaloblastic anemia secondary to folate deficiency. Different factors might cause folate deficiency anemia in pregnancy, including poor intake, impaired absorption and increased demand due to maternal erythropoiesis and fetal growth, arises mainly during the third trimester, particularly in untreated women.

Depending on the etiology of folate deficiency anemia in pregnancy, patients can benefit from the therapeutic effects of folate during pregnancy, from preconception until lactation. Clinicians usually determine the duration of folic acid treatment by close follow-up and laboratory investigations.

Pernicious anemia, which presents with similar clinical signs and symptoms as folate deficiency, needs to be well differentiated, as it requires B12 for management.

B12 is a water-soluble vitamin and its deficiency during the pregnancy is very rare due to large maternal storage, however, studies showed that B12 deficiency could also increase the risk of NTDs. As such, a rich diet could prevent such a deficiency and supplementation is rarely needed. Since folate supplement could mask the symptoms of B12 deficiency, the level of B12 should be measured before starting folic acid supplementation [46,47].

Serum folate below the normal range of 2.0–15 mg/l and red cell folate concentration below the normal range of 160–640 mg/l is diagnostic [48].

The effect on the fetus of folate deficiency anemia is not clear. The fetus has the ability to secure folic acid from the maternal circulation, even with maternal folate deficiency status, and can maintain stable hemoglobin and folate levels. At the same time, studies have detected fetal growth retardation [49] and smaller blood volume [50] as a consequence of pregnancy-induced megaloblastic anemia. Treatment involved the administration of oral folic acid, but the recommended dosage varies. In one respect, an oral intake of 0.5–1 mg two- or three-times daily intake is recommended as an adequate dose [51]. In another study, an oral 5 mg daily intake for 4 months is claimed to be a therapeutic dose [52].

ACOG states that a nutritious diet and folic acid and iron supplementation should be recommended to patients for treatment of pregnancy-induced folic acid deficiency. Treatment with 1 mg of oral folic acid daily typically produces an appropriate response [53].

## Folic acid & fetal growth restriction

Fetal growth restriction (FGR) is a term used to describe fetuses with an estimated fetal weight less than the tenth percentile for gestational age. Small for gestational age (SGA) is a term used exclusively to describe newborns whose birth weight is less than the tenth percentile for gestational age [54].

Fetal growth is dependent on maternal nutrition mainly in the preconception period and early pregnancy. Folate is critical in protein, DNA and lipid synthesis via the homocysteine pathway and is an essential factor for epigenetic mechanisms. Furthermore, while pregnancy progresses, folate demands and metabolism increase due to placental and fetal growth and development.

A positive correlation between maternal folic acid intake and fetal growth has been previously described, but most of those studies focused on the effect of folic acid on fetal growth in mid and late pregnancy [49,55–62]. A few studies have investigated this correlation in early pregnancy, but the results of these studies are not uniform [60,63].

A Cochrane review published in 2013 reported a positive association between folic acid supplementation and improvements in mean birth weight (BW), but no significant effect on BW less than 2500 g was noted [64]. In a systematic review by Fekete *et al*., the effect of folic acid supplementation on absolute BW was also observed [65]. They found a significant dose–response relationship between folate intake and birth weight. The Generation R study that was conducted in The Netherlands between 2002 and 2006, showed a positive association between preconception folic acid supplementation and higher BW and higher placental weights compared with no folic acid supplementation [66]. The range of folic acid intake in this study was 0.4–5 mg/day. A lower risk of low birth weight and SGA was also observed in women who took folic acid before conception than for women who did not take folic acid before conception. (OR: 0.43; 95% CI: 0.28–0.69) and (OR: 0.40; 95% CI: 0.22–0.72), respectively [66].

In a study, Hodgetts *et al*. included 11,736 women who had a singleton live birth with no congenital anomalies in the West midlands from 2009 to 2012 [67]. They reported that folic acid was associated with an SGA risk reduction if consumed preconception, but no significant risk reduction was found for postconception use. The range of folic acid intake in this review was from 0 to 5 mg daily. The highest rates of SGA occurred in women with no folate intake were 16.3 and 8.9% for the less than tenth percentile and the less than fifth percentile, respectively. Comparing preconception with postconception folic acid intake, the prevalence of BW less than tenth customized percentile was 9.9 and 13.8%, and the prevalence of BW less than fifth customized percentile was 4.8 and 7.1%, respectively. The conclusion was that folic acid could reduce the risk of SGA if used before conception [67].

The ACOG Practice Bulletin for Fetal Growth Restriction, mentions that although some dietary and nutritional supplemental strategies for SGA prevention have been studied, none of these strategies were found to be effective and are not recommended [54].

## Folic acid & autism

Autism spectrum disorders (ASDs) are neurodevelopmental disorders characterized by social deficits, communication difficulties and repetitive or stereotyped behaviors [68]. The prevalence is approximately 1% among children [69].

Recently, reducing the risk of autism by folic acid intake and the time of its consumption have been closely investigated [70,71]. The optimal protective effect of folic acid in preventing autism is achieved when folic acid is taken preconceptionally and in early pregnancy because this is the critical period for brain development and development of neurologic pathologies such as NTDs. Further research is warranted to investigate the effect of folic acid on autism if it is used in late pregnancy.

A combination of genetic [72,73] and environmental [74] factors contributes to ASD development. Folic acid has been hypothesized to play a beneficial role in ASD prevention. Initially, Roth *et al*. performed a prospective Norwegian Mother and Child Cohort Study (MoBa) [70]. They determined that preconception folic acid supplementation (4 weeks before to 8 weeks after conception) is associated with a lower risk of severe language delays among 3-year-old children. Suren *et al*. selected a sample size of 85,176 children from the MoBa study [71]. They included children who were born in 2002 through 2008 and followed them through 2012. The mean age of the children at follow-up was 6.4 years. Among them, 114 children were diagnosed with autistic disorder. They found a beneficial role of folic acid supplementation on ASD rate. The dosage of folic acid in the MoBa study was 200–400 μg/day, which is the common multivitamin and prenatal supplement dose in Norway [71]. In Nepal, Christian *et al*. followed 676 children aged 7–9 years from June 2007–April 2009 [75]. The children's mothers participated in a randomized controlled trial of prenatal and postnatal micronutrient supplementation. This trial was conducted in a rural area of Nepal, where iron deficiency is prevalent. The study found a positive association between prenatal iron/folic acid supplementation and different aspects of intellectual functioning, including memory, inhibitory control and fine motor functioning, among offspring. The dosage of folic acid was 400 μg/day [75]. Schmidt *et al*. performed a large population-based case-controlled study in CHARGE from 2003 to 2009 [76]. They recruited families with a child diagnosed with ASD, DD (developmental delay) or TD (typical delay). In the CHARGE study, the association between folic acid and reduced risk of ASD was more significant for mothers and children with the MTHFR 677 C>T variant genotype. MTHFR 677 C>T is a genetic polymorphism that is associated with high homocysteine; therefore, fetuses with this genotype require higher amounts of folate for appropriate neurodevelopment. The folic acid intake was 0–800 μg/day. The results showed that a mean daily folic acid intake of greater than 600 μg as compared with less than 600 μg during the first month of pregnancy was associated with a reduced risk of ASD (adjusted OR: 0.62; 95% CI: 0.42–0.92: p = 0.02) [76].

Another study, worth to be mentioned here is the Neurodevelopmental Effects of Antiepileptic Drugs [77] study. They reported cognitive outcomes on 309 mother/child pairs exposed to AED monotherapy: CBZ, Lamotrigine (LTG), Phenytoin (PHT) and VPA. The primary outcome was IQ at 6 years, adjusted for maternal IQ, AED type, AED standardized dose, gestational age and use of periconceptional folate. Results indicated that the VPA group had statistically significant lower IQ scores compared with those taking the other three AEDs at 3 and 6 years follow-up, with a more prominent decline in verbal scores [77,78]. The effects on IQ along with verbal ability, nonverbal ability, memory and executive function are also observed to be dose dependent, although no particular dose of VPA could be deemed as a safe range [77].

Use of periconceptional folate along with any AED interestingly showed better mean IQ scores within the same AED group, thus re-emphasizing the early supplementation of folic acid; consistent with recent beneficial role of folic acid in cognitive function and ASD prevention. Further studies are warranted to prove the potential benefit of folic acid in preventing ASD.

## Timing

The timing of folic acid supplementation is crucial. The beneficial timing of folic acid intake on different pregnancy outcomes is summarized in Table 5.

## Adverse effects

Folic acid is generally considered to be a safe supplement. Its toxicity and adverse effects have been studied across a wide range of dosages and in different populations. In a review article that summarized the literature between 1966 and 1994, the potential side effects of folic acid were listed to be difficulty in ruling out B12 deficiency, interaction with drugs that inhibit folate metabolism, decreased zinc absorption, hypersensitivity reactions, association with malignancy, neurotoxicity and epileptogenic effects and increased susceptibility to malaria [79]. Weight loss, gastrointestinal, neurologic and psychiatric side effects were observed in an unblinded, uncontrolled trial after a month of taking 5 mg, three-times/day folic acid, which lead to study cessation [80]. Moreover, these effects were not observed in randomized, double–blind, placebo-controlled studies with the same dose of folic acid for 4 weeks [81,82].

The above-mentioned studies were done on nonpregnant patients; and in this review we mainly focus on maternal folic acid supplementation in an obstetric population.

The clinical manifestations of normal pregnancy are very variable, and some of the mentioned folic acid side effects, such as hypersensitivity reactions and gastrointestinal symptoms, could be attributed to normal pregnancy symptoms. Beyond these subtle side effects, a few adverse effects of folic acid on offspring and mothers should be noted herein.

## Asthma/allergy in offspring

Haberg *et al*. determined that maternal folic acid supplementation could increase the relative risk of lower respiratory tract infections and wheezing at 6–18 months of age, especially if it is consumed during the first trimester [83]. In an Australian prospective cohort study, (n = 557), Withrow *et al*. focused more on the timing and dosage of maternal folic acid intake and its effect on childhood asthma [84]. They found that folic acid supplementation in late pregnancy increases the risk of asthma in children at 3.5 years as well as persistent asthma. Additionally, intake of greater than 400 μg folic acid daily might cause repeated presence of unmetabolized folic acid in maternal and fetal circulation, especially in countries with fortified foods, but the effect of these unmetabolized analogs is not clear [85]. Dunstan *et al*. showed that folic acid supplement greater than 500 μg/day was associated with an 85% greater risk of allergic diseases compared with intake of 200 μg/day [86]. In contrast, other studies have not supported the above-mentioned findings. In a study by Magdelijns *et al*., no association between maternal folic acid intake and wheezing or asthma was found in children aged 6–7 years [87]. Furthermore, Martinussen *et al*. reported no correlation between maternal folic acid intake in early pregnancy and asthma in the children at 6 years [88].

## Folic acid & malignancy

Folic acid has shown a dual effect regarding malignancy, both a protective effect and a carcinogenetic effect. Few studies have described an inverse relationship between dietary folate intakes and cervical neoplasia [89]. Charles *et al*. found a slight increase in the risk of breast cancer in pregnancy by comparing a low dose (0.2 mg) and high dose (5 mg) of folic acid [90]. On the other hand, in a large cohort study, Zhang *et al*. did not find a correlation between the overall risk of breast cancer and folic acid intake [91]. They noted that a higher dose of folate or multivitamin could reduce the risk of breast cancer among women who consume alcohol.

In addition, there have been concerns regarding folic acid supplementation and increasing colon cancer risk. The absolute rates of colorectal cancer (CRC) began to increase in 1996 (USA) and 1998 (Canada), peaked in 1998 (US) and 2000 (Canada), and have continued to exceed the pre-1996/1997 trends by four to six additional cases per 100,000 individuals. In each country, the increase in CRC incidence from the prefortification trend falls significantly outside of the downward linear fit based on nonparametric 95% confidence intervals. The observation did not prove causality, but it was hypothesized that the institution of folic acid fortification may have been wholly or partly responsible for the observed increase in CRC rates in the mid-1990s [92].

Following the dual protective versus carcinogenic potential effect of folic acid, a double blind, placebo-controlled, randomized clinical trial between 1994 and 2004, was conducted to assess the safety and efficacy of folic acid to prevent colorectal adenomas. They concluded that 1 g/day dose of folic acid does not decrease the colorectal adenoma risk [93].

At this time, there are not enough data to support a significant causal effect of folic acid on malignancy, and further research in this area is clearly warranted.

## Folic acid & twinning

It is still uncertain whether a higher dosage of folic acid could increase the risk of twinning. In a Hungarian randomized controlled trial (RCT), Cetizel and Dudás found a 40% increase in multiple births among women who took multivitamin supplementation with 0.8 mg of folic acid [94]. In the Hungarian Case Control Surveillance of Congenital Abnormalities (HCCSCA), which was conducted from 1980 to 1996, women were categorized into three subgroups: those who took pre- and post-conception folic acid supplementation (6 mg in general), those who took multivitamins containing 0.1–1.0 mg folic acid and those who consumed folic acid plus a multivitamin [95]. The three subgroups’ outcome was compared with the outcome of an unsupplemented control group. There was an association between pre- and post-high-dose folic acid and multivitamin supplementation and a mild increase in twin pregnancies. Using the Swedish Medical Birth Registry, Ericson *et al*. found an increase in the rate of twin deliveries among women who took folic acid in early pregnancy [96]. However, in a large-scale cohort study in China, among almost a quarter million woman, the rate of twin pregnancies was similar between those who took 0.4 mg of preconception folic acid supplement and the control group [97]. Currently, there is no consistent evidence that supports the effect of preconception folic acid or fortification on twin pregnancy.

## Conclusion

The effect of folic acid in preventing NTDs is very convincing, and international guidelines recommend it as a preconception supplement in different dosage according to the baseline risk. The beneficial role of folic acid on pregnancy outcomes, mainly in gestations complicated by seizure disorders, preeclampsia, anemia, fetal growth restriction, as well as its role in modulating the fetal origins of autism have been controversial. In that aspect, folic acid in general and its dosage in particular are still debatable. Overall, the benefit of folic acid outweighs the theoretical risks, as of those risks were not proven.

In conclusion, folic acid use to prevent adverse pregnancy outcomes, beyond NTD prevention, is a great area of interest and future studies are warranted to support its clinical use and value.

## Future perspective

We speculate that in the next decade folic acid mechanisms of action would be further understood, and with the advent of the genetic revolution, targeted therapy would prevail rather than universal supplementation. That therapy could be through an adjusted dose for at-risk pregnant women or fetuses, or through targeted transplacental delivery using nanomedicine technologies.

**Table 1.** **International Guidelines for Preventive Folic Acid Dosage in Pregnancy American Congress of Obstetrics and Gynecology Recommendation.**

**Level of evidence**	**Low-risk women**	**400 μg/day**
Level A^†^	High-risk women/women who have had NTD in previous pregnancy	4 mg/day
	MSAF evaluation is an effective screening test for NTDs and should be offered to all pregnant women	
Level B^‡^	Women with elevated serum AFP	Should have a specialized ultrasound examination to further assess the risk of NTDs
	Fetus with an NTD	Should be delivered at a facility that has personnel capable of handling all aspects of neonatal complications
Level C^§^	Women capable of becoming pregnant	400 μg/day
	The ideal dose of folic acid has not been appropriately evaluated in prospective clinical trials	
	Route of delivery for the fetus with an NTD	Should be individualized due to the lack of data indicating that any one dosage provides a superior outcome

^†^Level A: The following recommendations are based on good, consistent scientific evidence.

^‡^Level B: The following recommendations are based on limited or inconsistent scientific evidence.

^§^Level C: The following recommendations are based primarily on consensus and expert opinion.

AFP: Alfa fetoprotein; MSAF: Maternal serum alfa fetoprotein; NTD: Neural tube defect.

Data taken from [14].

**Table 2.** **Royal College of Obstetrics and Gynecologists Recommendation.**

**All pregnant women**	**400 μg/day preconception until 12th week of pregnancy**
High-risk pregnant women^†^	5 mg/day

^†^High-risk women:

– History of neural tube defect in previous pregnancy

– History of neural tube defect in pregnant woman or in her partner

– Using certain epilepsy medications

– Diabetes or celiac disease

– BMI 30 or more

– Sickle cell anemia or thalassemia

Data taken from [15,16].

**Table 3.** **Society of Obstetricians and Gynecologists of Canada Recommendation.**

**Risk category**	**Recommendation**
Low risk group^†^	0.4 mg/day: beginning at least 2–3 months before conception, continuing throughout the pregnancy and for 4–6 weeks postpartum or as long as breastfeeding continues
Moderate risk group^‡^	1.0 mg/day: beginning at least 3 months before conception, continuing until 12 weeks’ gestational age. From 12 weeks, throughout the pregnancy, and for 4-6 weeks postpartum or as long as breastfeeding continues, daily multivitamin supplementation with 0.4–1.0 folic acid is recommended
Increased/high risk group^§^	4.0 mg/day: beginning at least 3 months before conception, continuing until 12 weeks’ gestational age. From 12 weeks’ gestational age, continuing throughout the pregnancy, and for 4-6 weeks postpartum or as long as breastfeeding continues, daily multivitamin supplementation with 0.4–1.0 folic acid is recommended

Personal positive or family history of other folate-sensitive congenital anomalies (limited to specific anomalies for cardiac, limb, cleft palate, urinary tract, congenital hydrocephaly).

Family history of NTD in a first or second-degree relative. Maternal diabetes (type I or II) with secondary fetal teratogenic risk. Measurement of red blood cell folate levels could be part of the preconception evaluation to determine the multivitamin and folic acid supplementation dose strategy (1.0 mg with RBC folate< 906 and 0.4 to 0.6 mg with RBC folate >906) with a multivitamin).

Teratogenic medications with secondary fetal teratogenic effects by folate inhibition via anticonvulsant medications (carbamazepine, valproic acid, phenytoin, primidone, phenobarbital), metformin, methotrexate, sulfasalazine, triamterene, trimethoprim (as in cotrimoxazole), and cholestyramine.

Maternal GI malabsorption conditions secondary to co-existing medical or surgical conditions that have been shown to result in decreased RBC folate levels (Crohn's or active Celiac disease, gastric bypass surgery, advanced liver disease, kidney dialysis, alcohol overuse).

^†^LOW risk group: women or their male partners with no personal or family history of health risks for folic acid sensitive birth defects.

^‡^MODERATE risk group: women with the following personal or co-morbidity scenarios (1–5) or their male partner with a personal scenario (1 and 2).

^§^INCREASED/HIGH risk group: women or their male partners with a personal NTD history or a previous neural tube defect pregnancy.

Data taken from [17].

**Table 4.** **International Recommendations for Folic Acid in Pregnant Women with Epilepsy.**

**Society**	**Recommendation**	**Ref.**
ACOG	4 mg/day	[26]
NICE	5 mg/day	[27]
SOGC	5 mg/day	[28]

**Table 5.** **Folic acid supplementation timing and pregnancy outcomes.**

**Outcome/time**	**Preconception**	**First trimester**	**Second trimester**	**Third trimester**
NTD	^‡^	^‡^	^§^	^§^
Preeclampsia	^†^	^†^	^‡^	^†^
Anemia	^†^	^†^	^†^	^†^
FGR	^‡^	^‡^	^†^	^†^
Autism	^†^	^†^	^§^	^§^

^†^Beneficial.

^‡^Significant benefit.

^§^No benefit.

FGR: Fetal growth restriction; NTD: Neural tube defect.

**Executive summary**

Folic acid plays a beneficial role in pregnancy, and its association with fetal outcomes has been widely studied.
Evidence shows that folic acid supplementation could modulate other adverse pregnancy outcomes, such as seizures in women known with epilepsy or seizure disorders, preeclampsia, pregnancy-induced anemia, autism, fetal growth restriction, preterm delivery and congenital birth defects other than neural tube defects.
Current recommendations from American College of Obstetricians and Gynecologists, Royal College of Obstetricians and Gynecologists and Society of Obstetricians and Gynecologists of Canada are presented in this review.
The effect of folic acid in preventing neural tube defects (NTDs) is very significant, and most international guidelines recommend it as a preconception supplement.
Folic acid supplementation is an important part of the prenatal care of women with epilepsy mainly due to interference of antiepileptic drugs in folate metabolism.
Folic acid could correct hyperhomocysteinemia by optimizing the homocysteine pathway, thus can play a role in reduction of the incidence of gestational hypertension or preeclampsia.
Depending on the etiology of folate deficiency anemia in pregnancy, patients can benefit from the therapeutic effects of folate during pregnancy, from preconception until lactation. American College of Obstetricians and Gynecologists states that a nutritious diet and folic acid and iron supplementation should be recommended to patients for treatment of pregnancy-induced folic acid deficiency. Treatment with 1 mg of oral folic acid daily typically produces an appropriate response.
Reducing the risk of autism by folic acid intake is under study especially in early pregnancy within the critical period for brain development and development of neurologic pathologies such as NTDs.
Folic acid is generally considered to be a safe supplement. The potential side effects of folic acid were listed to be difficulty in ruling out B12 deficiency, interaction with drugs that inhibit folate metabolism, decreased zinc absorption, hypersensitivity reactions, association with malignancy, neurotoxicity and epileptogenic effects and increased susceptibility to malaria.
Folic acid use to prevent adverse pregnancy outcomes, beyond NTD prevention, is a great area of interest and future studies are warranted to support its clinical use and value.
